# Interference of tonic muscle activity on the EEG: a single motor unit study

**DOI:** 10.3389/fnhum.2014.00504

**Published:** 2014-07-11

**Authors:** Gizem Yilmaz, Pekcan Ungan, Oğuz Sebik, Paulius Uginčius, Kemal S. Türker

**Affiliations:** ^1^Koç University School of MedicineSariyer, Istanbul, Turkey; ^2^Institute of Physiology and Pharmacology, Medical Academy, Lithuanian University of Health SciencesKaunas, Lithuania

**Keywords:** electromyogram, electroencephalogram, muscle artifact, single motor unit, interference

## Abstract

The electrical activity of muscles can interfere with the electroencephalogram (EEG) signal considering the anatomical locations of facial or masticatory muscles surrounding the skull. In this study, we evaluated the possible interference of the resting activity of the temporalis muscle on the EEG under conventional EEG recording conditions. In 9 healthy adults EEG activity from 19 scalp locations and single motor unit (SMU) activity from anterior temporalis muscle were recorded in three relaxed conditions; eyes open, eyes closed, jaw dropped. The EEG signal was spike triggered averaged (STA) using the action potentials of SMUs as triggers to evaluate their reflections at various EEG recording sites. Resting temporalis SMU activity generated prominent reflections with different amplitudes, reaching maxima in the proximity of the recorded SMU. Interference was also notable at the scalp sites that are relatively far from the recorded SMU and even at the contralateral locations. Considering the great number of SMUs in the head and neck muscles, prominent contamination from the activity of only a single MU should indicate the susceptibility of EEG to muscle activity artifacts even under the rest conditions. This study emphasizes the need for efficient artifact evaluation methods which can handle muscle interferences.

## Introduction

The origin of the EEG signal is still a popular question in the broad neuroscience research area. From daily clinical use to the sophisticated brain computer interface (BCI) systems, recognition and the handling of artifacts have gained much more importance in recent years. Among biological artifacts originating from a subject's body (myogenic, eye blink, ocular, cardiovascular), the myogenic contamination of the EEG constitutes a serious problem. In fact, many studies have reported that the EMG signal originating from facial or masticatory muscles can contaminate the EEG (O'Donnell et al., 1974; van de Velde et al., 1998; Goncharova et al., 2003; Fu et al., 2006; ter Meulen et al., 2006; Fatourechi et al., 2007; Whitham et al., 2007; Ma et al., 2012). Since EEG electrodes are placed on or around the cranial muscles, the myogenic activity of the frontalis, temporalis (Goncharova et al., 2003; Fu et al., 2006; Yong et al., 2008), ocular muscles (Kovach et al., 2011; Carl et al., 2012; Nottage et al., 2013), neck muscles (Whitham et al., 2007, 2008), and the peri-auricular muscles (ter Meulen et al., 2006) may interfere with the recorded EEG signal.

The main reason for this contamination is the crosstalk between nearby recording sites. Conventional EEG filters, which are mostly set to 0.1 Hz high-pass and 70 Hz low-pass (Niedermeyer, 1999) are insufficient to reduce the EMG interference as the frequency spectra overlap between the EEG and the EMG signals cover a broad range including the alpha (8–13 Hz), beta (14–30 Hz), and gamma (above 30 Hz) bands (Friedman and Thayer, 1991; Türker, 1993; Akay and Daubenspeck, 1999; Goncharova et al., 2003; Fu et al., 2006). However, this inadequacy of ordinary filtering is compensated by several other artifact removal methods such as spatial filtering (McFarland et al., 1997; Fitzgibbon et al., 2013), adaptive filtering (Boudet et al., 2012) and component based algorithms such as Independent Component Analysis (ICA) (Jung et al., 2000; Olbrich et al., 2011), Principal Component Analysis (PCA), or Canonical Component Analysis (CCA) (De Clercq et al., 2006). Ideally, any of these correction and removal techniques should possess a high degree of sensitivity and specificity which means that while attenuating muscle artifacts, neurogenic signals must be preserved.

To illustrate the interference between the EMG and EEG signals researchers asked subjects to voluntarily contract their (facial or masticatory) muscles during EEG recording sessions (O'Donnell et al., 1974; Friedman and Thayer, 1991; Goncharova et al., 2003; Fu et al., 2006; Yong et al., 2008). The reported high degree of contamination was not surprising due to crosstalk from the neighboring muscles/sources of bioelectrical potentials (Türker and Miles, 1990).

In routine EEG recordings, the subject is instructed to sit or lie down, relax and only to attend to the signs/suggestions that appear on a monitor. Depending on the purpose of the study, the subject can passively read a book/watch a muted film with subtitles to make him/her ignore any stimulus and avoid drowsiness. Under these “relaxed” recording conditions, many neck, mimic and masticatory muscles are unintentionally activated to keep the head up (Kumar et al., 2003; Siegmund et al., 2007); the mouth closed (Møller, 1976; Woda et al., 2001) the eyes open, and the facial gesture expressed (Sumitsuji, 1986; Waterink and van Boxtel, 1994; Dimberg et al., 2000). Therefore, the interference of this unintentional muscular activity with the EEG signal may go unnoticed. A few recent studies show that the EEG was contaminated by the low level activities of the subjects' scalp and neck muscles (Goncharova et al., 2003; Ma et al., 2012; Nottage et al., 2013). However, studies to date have not assessed the contribution of single motor unit (SMU) action potentials to EEG records. SMU potentials are all-or-nothing events and their muscular source can be obtained with confidence. Furthermore, a study on the interference of SMU activity in relaxed position appears to be an urgent need because the “EEG” signal is used to indicate cortical activity and also to check the status of coma (Sethi et al., 2008). Considering the fact that various narrow-band frequency components are interpreted in the EEG literature as electrical oscillations with a cortical origin, possible myogenic contributions to EEG emphasizes an important problem.

In this study we aimed to explore the extent of the EEG contamination induced by the firing of a single motor unit. Since a low level of tonic activity is known to exist in the temporalis muscle at rest (Møller, 1976) which keeps the mandible in the physiological rest position 2–3 mm between the upper and lower incisors (Nairn, 1976), the primary objective is to find out the existence of the contribution of spontaneously active temporalis SMUs onto the EEG signal. The secondary objective is to determine the distribution profile of the SMU interference on the EEG signal.

## Methods

### Subjects

Data were collected from 9 healthy male volunteers (aged 20–42 years). All volunteers gave written consent for the experimental procedures. The study was approved by the Koç University Local Ethics Committee. All subjects were right handed, none had temporomandibular joint disorders.

### Experimental setup

EEG activity was recorded with the 10–20 system EEG Headcap (MEDCAP, Spes Medica S.r.l, Italy) with 21 Ag/AgCl electrodes. The electrodes Fp1, Fp2, Fz, F3, F4, F7, F8, C3, C4, T3, T4, T5, T6, Pz, P3, P4, O1, O2, all referenced to Cz, were used. The clip electrode for grounding was attached to the right ear lobe and electrode impedances were kept below 20 kOhm by filling the electrode-tissue interface with conductive gel (Electro-Gel, ECI, USA).

**Figure 1:**
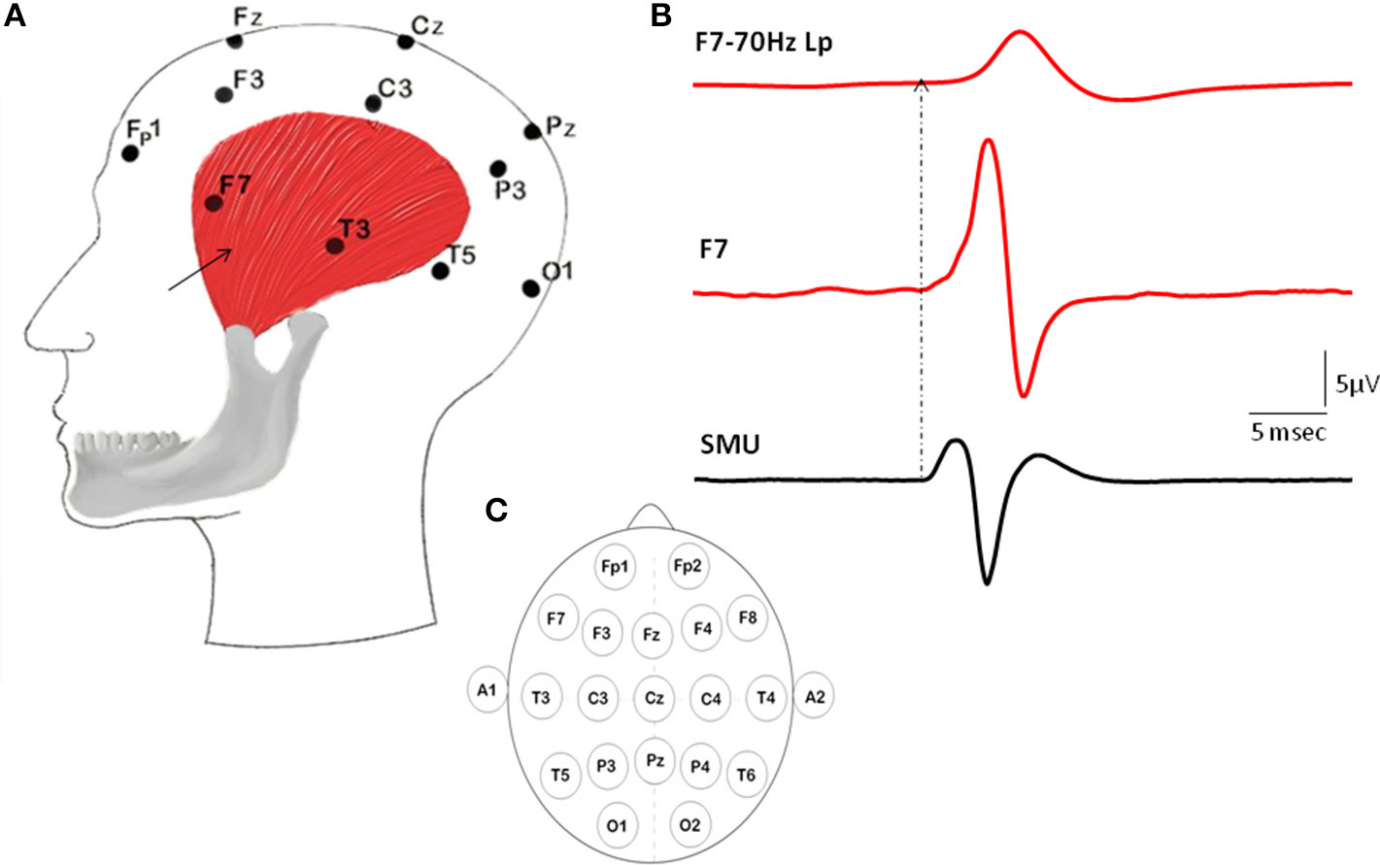
**Illustration of the locations of intramuscular electrodes, EEG electrodes, Macro-EMEG, and SMU potential. (A)** Intramuscular wire is depicted as a black arrow placed on the anterior temporalis. **(B)** Represents a Macro-EMEG potential from the F7 electrode with (top) and without (middle) low pass filtering and single motor unit (SMU) action potentials recorded intramuscularly from the temporalis muscle that are used as triggers (bottom). **(C)** Illustrates the EEG electrode positions on the scalp.

SMU activity was recorded using two intramuscular silver fine-wire electrodes coated with Teflon (75 μm in core diameter; Medwire, USA). The tips of the wires were stripped off their Teflon coating about 3 mm to record the electrical activity of motor units from a bigger volume. The medial border of the left temporalis was detected by palpation and a 25 G surgical needle with the pair of wires inside was inserted in the 1–2 cm above of the midline of zygomatic arch into the relaxed muscle. The needle was immediately withdrawn leaving “fish-hooked” electrodes within the muscle (Figure 1).

During recordings subjects were seated comfortably in an armchair inside an isolated EEG chamber with a Faraday Cage. They were instructed to relax unless asked by the experimenter to make specific facial movements. Their heads were unsupported. After placing the EEG cap and inserting the indwelling pair of wire electrodes, EEG and SMUs activity were recorded concurrently. The same SMUs were followed for 4–5 min to have enough number of motor unit discharges for the averaging purposes.

A typical experiment with a specific SMU consisted of three EEG-SMU recording trials: relaxed eyes open, relaxed eyes closed, and relaxed dropped jaw. The same procedure was followed for each spontaneously active SMU. The EEG epochs containing voluntary muscle contraction such as clenching the teeth, swallowing, shutting the eyes tight and raising the eyebrows were excluded from the data.

EEG-SMU data were collected with the same software (SystemPlus, Micromed S.p.A, Italy) and amplifier (Micromed S.p.A, Italy). The sampling rate was 4096 Hz, and filters were set to 0.15 Hz high pass and 1500 Hz low pass. A 100 Hz HP filter was applied to the SMU channel only for monitoring the unit potentials with ease during the experiment.

### Analysis

The recorded SMU and EEG data were exported to Spike2 (Cambridge Electronic Design, England) for further analysis. SMU channel was high-pass filtered at 100 Hz. Template matching decomposition analysis program (Spike 2) identified SMU action potentials. Identified SMU action potentials were used as triggers and EEG signals as source in a spike triggered averaging (STA) procedure to assess the amount of EMG interference within 50 ms around the triggers (Figure 1).

The STA process resulted in different amplitudes of interference potentials, which were referred in this study as Macro-EMEG (electro-myo-encephalogram). Cz-referenced potentials were converted into reference free Macro-EMEG by using Average Reference (AR) Method (Dien, 1998). The calculated mean of all EEG channels was subtracted from each channel.

Global field power (GFP, Lehmann, 1987) analysis determined the latency information of Macro-EMEG potentials. Peak latency of Macro-EMEG potentials is determined as the latency of the global field power (GFP, Lehmann, 1987) maximum. Macro-EMEGs were normalized as the percentage of the greatest Macro-EMEG amplitude to compare the spatial distribution.

## Results

Seventeen different SMUs were identified from the 6 subjects in whom we detected spontaneous SMU activity. In three subjects however no SMU activity was recorded at rest and therefore EEG data from these subjects were excluded from this study. When active at rest, the same motor unit had multiple epochs of discharge during the experiment. Each episode of continuous discharge was treated as a new SMU-train. Respectively, 39 SMU-trains were used as triggers for the STA process (Table 1). The mean discharge frequency was 15.9 ± 3.6 Hz for all records. Thirty one SMU-trains belonging to 12 unique SMUs generated Macro-EMEG potentials and were used for further calculations. Contributions of five SMUs on the EEG were not notable as shown in Table 1. Note that the eyes open condition was over represented in the Table since in the eye close and jaw drop conditions some of the previously active units stopped to fire. Therefore, eye close (C) jaw drop (J) conditions were under represented in the Table.

**Table 1:** **Condition and frequency information of SMUs**.

**SMU**	**Condition**	**Discharge Freq (Hz)**	**SMU**	**Condition**	**Discharge Freq (Hz)**	**SMU**	**Condition**	**Discharge Freq (Hz)**
1	EO	16.8	7	EO	18.0	1	EO	10.5
	EO	15.5		C	16.0		EO	13.6
	EO	13.3	8	EO	22.4		EO	11.3
2	EO	15.2		EO	22.7	2	C	19.1
	C+J	12.6		EO	22.4		C	22.7
	EO	14.4		C	20	3	EO	17
3	EO	16.8		EO	22.1	4	EO	17
	C	15.3	9	C	11	5	EO	19.8
	EO	16.2		C	18.4			
4	EO	11.5		EO	16.7			
	EO	12.2	10	EO	18			
	EO	13.4		EO	11.9			
	EO+J	13.7		C	10.5			
	C	13.4	11	EO	17.6			
5	EO	16.1	12	EO	11.7			
6	C	14.8						

SMUs in left and middle columns generated prominent Macro-EMEGs, SMUs in right column were not represented in Macro-EMEG (EO, Eyes open; C, eyes closed; J, jaw dropped).

Note that the discharge rates could be given only when there was a single motor unit activity during a predetermined condition. Therefore, jaw drop (J) condition, where most of the existing single unit activity stopped, was under represented in the Table.

**Figure 2:**
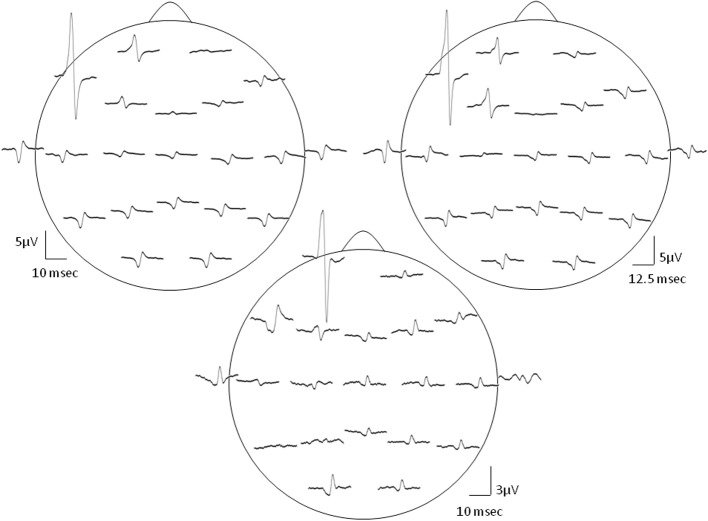
**Distribution of the SMU representation in various EEG electrodes.** Topography of the Macro-EMEG waveforms of 3 different SMUs is depicted in this figure. In the top two depictions, the largest Macro-EMEG was in electrode F7 while in the bottom depiction the Macro-EMEG was the greatest in electrode Fp1 due probably to the proximity of the recorded SMU and its muscle fiber orientation.

When SMU spikes of temporalis muscle were used as triggers for the STA process, SMUs generated prominent Macro-EMEG potentials on the ipsilateral-frontal and ipsilateral-parietal locations of the EEG. The F7 electrode was located just above the area where intramuscular electrodes recorded SMU potentials (Figure 1). The amplitude of the Macro-EMEG potential in the F7 electrode was the largest in 10 of the 12 SMUs and therefore its size was used as the normalization factor for pooling the contribution of these 10 SMUs (Figure 2).

**Figure 3:**
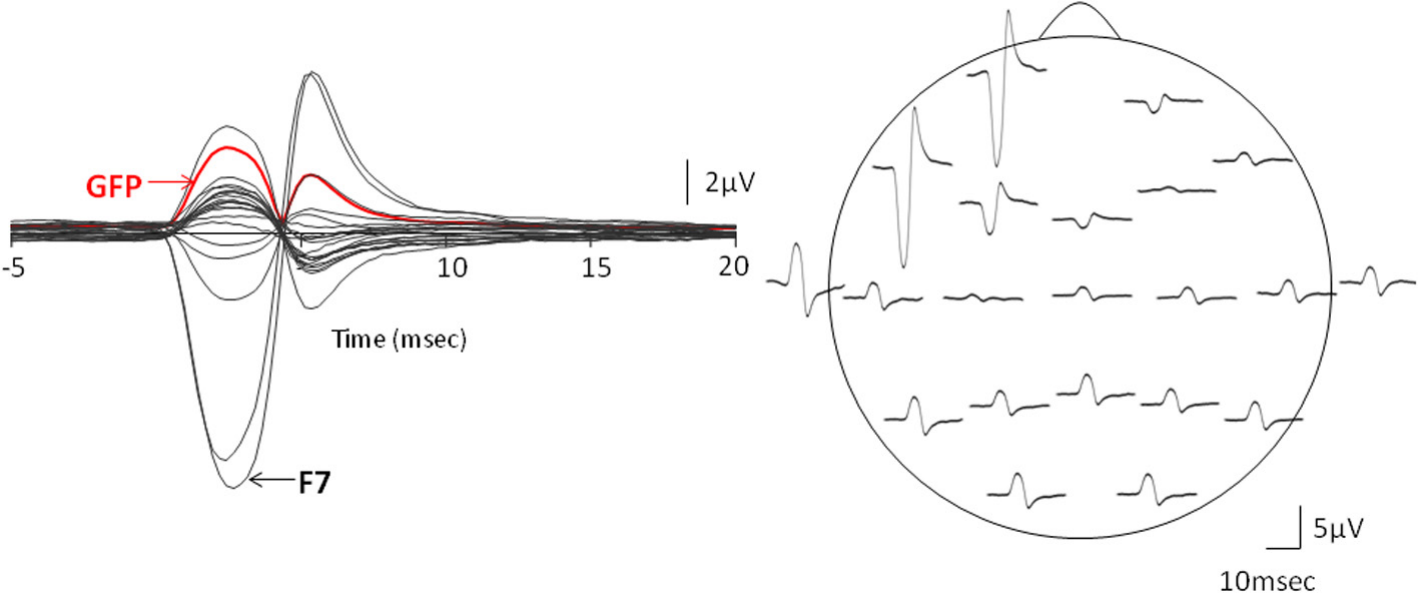
**Evidence for the cross-talk.** The left column presents the global field power (GFP) vs. time plot of the Macro-EMEG potentials from the 21 EEG electrodes and the right column shows the topography of Macro-EMEGs of the same SMU. Clear overlapping shows the occurrence of the potentials without a time delay, indicating cross-talk. Again, F7 electrode had the greatest interference.

Figure 2 demonstrates the widespread interference of the resting temporalis SMU activity on the EEG signal for 3 SMUs. EEG records obtained from the electrodes closer to the SMU were contaminated the most. In particular, frontal electrodes F7, Fp1, F3 showed pronounced Macro-EMEG potentials indicating strong contribution of SMU activity on the EEG signal. Ten of 12 SMUs had the biggest Macro-EMEG at F7 and 2 SMUs at the Fp1 electrode (Figure 2 top and bottom figures). Contamination was not limited to frontal electrodes. Interference of one SMU was evident in other electrodes, which were far from the SMU. However, for some electrode locations a clear potential was not observed after averaging. An example of this is shown in electrodes T5, P3, and A2 (Figure 2 lower section). Nonetheless, the ipsilateral side of the head was found to be more susceptible compared to the contralateral side in terms of the amplitude of the Macro-EMEG potentials. A clear phase reversal of the biphasic Macro-EMEG was observed across the coronal line (Figures 2, 3). Polarity of the Macro-EMEGs changed due to the orientation of dipolar sources. Bottom section in Figure 2 shows the polarity change between Fp1 and F7 while the polarity was the same in the upper two sections.

Macro-EMEG potentials occurred synchronously over the skull. Figure 3 compares the GFP with time course of the Macro-EMEG potentials recorded from the 21 electrodes. The left column shows the overlapping of Macro-EMEGs and GFP while the right column details the topography and phase reversal of Macro-EMEGs for the same SMU (Figure 3). There was no time delay between the occurrences of Macro-EMEG potentials indicating that the interference is a volume-conducting cross-talk event which does not involve neuronal conduction (Figure 3).

**Figure 4:**
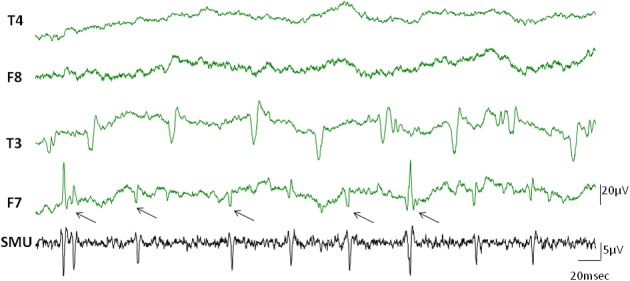
**Visible SMU spikes in EEG records.** Clearly visible cross-talks of the SMU spikes occasionally appeared on the EEG channels during recording. Black arrows indicate cross-talks of the simultaneously recorded SMU spikes in the F7 electrode. Other clear spikes in the F7 and T3 electrodes may however belong to different simultaneously active motor units which were not in the recording range of our wire electrodes.

Occasionally, discharges of single motor units occurred in the EEG channels simultaneously in the form of visible spikes (Figure 4). In this example while the temporalis SMU activity was the source of the spikes in F7 electrode of the EEG, the T3 channel represented the activity of another active SMU. The electrodes in the contralateral side did not show visible SMU spikes for this particular epoch.

**Figure 5:**
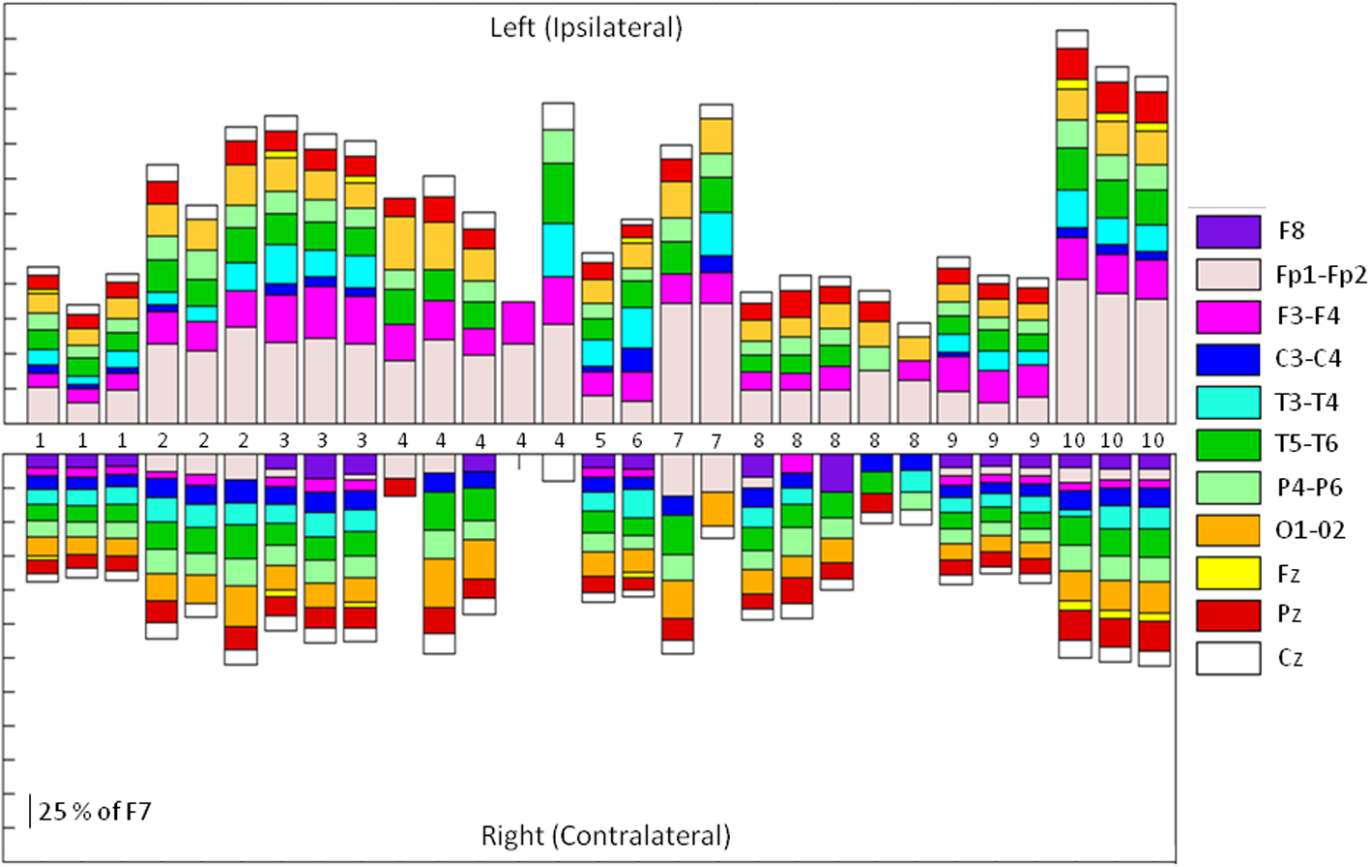
**Relative sizes of the SMU representation on different EEG electrodes.** Stacked bar representation of the percentage contribution of 29 SMU-trains to Left and Right Side EEG electrodes. Each of the 29 SMU trains is represented as a bar in horizontal axis. Macro-EMEG representation in electrode F7 was taken as 100 percent and SMU representations in all other electrodes were shown as a percentage of F7. Since the SMU representation in F7 is used for normalization (100 percent), it was not depicted in the figure. Numbers in the middle indicate the unit numbers and the number of times the same unit was used in different epochs of the experiment.

Thirty one SMU-trains belonging to 12 SMUs produced Macro-EMEG potentials. To point out the differences in the contribution of each SMU train to each of the EEG channels, the size of the Macro-EMEGs in all channels were normalized to the size in the F7 electrode for the 29 SMU trains (Figure 5). Two SMUs which had the largest Macro-EMEG size in the Fp1 electrode were not included in Figure 5.

**Figure 6:**
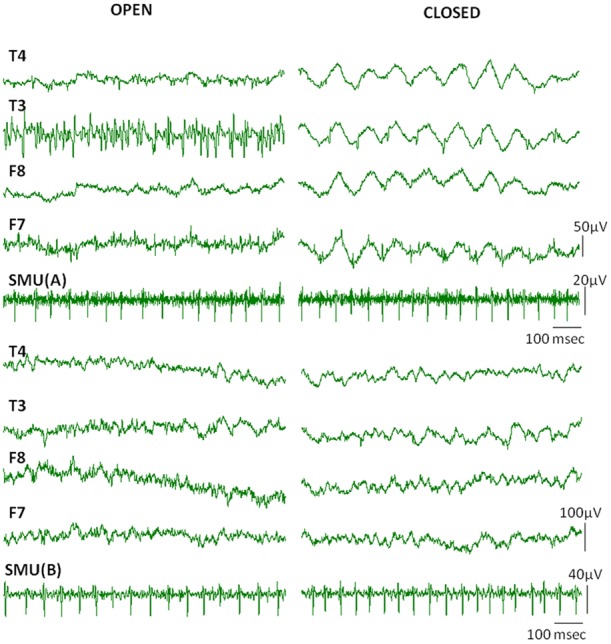
**Effect of closing the eye.** Comparison of visible EMG interference on EEG channels when eyes are open (left) and closed (right) in a relaxed condition. The silencing effect of the eyes closed procedure is clearly seen in EEG but not in the SMU activity. This is because the muscles which ceased activity in eyes closed condition were not the ones from which the SMU activity was recorded.EEG electrodes F7, F8 and T3, T4 were located on anterior and medial temporalis respectively (Also refer to 1C for electrode names).

When subjects were instructed to close their eyes some active SMUs silenced. Nine of the 17 SMU trains continued to be active even after closing the eyes (Table 1). Although the high frequency activity on the EEG signal diminished in the eyes closed condition, the Macro-EMEG amplitude did not change when the SMU continued its activity under the eyes closed state. Figure 6 compares two experimental conditions (eyes open and eyes closed) for visible SMU activity on EEG channels. Two different SMUs in Figure 6 generated similar Macro-EMEG amplitudes in both states.

Subjects were also asked to “drop their jaws” to stop the temporalis from holding the mandible. Only two units continued to fire after jaw dropping but other SMUs stopped firing. This observation suggests that the jaw drop procedure may be useful for reducing the EMG interference of temporalis origin in EEG records.

## Discussion

In this study we investigated the possible interference of the resting activity of muscles around the head on EEG records. We used the resting activity from the SMUs of the temporalis muscle as the trigger and averaged the simultaneously recorded electrical activity from the EEG electrode using a STA procedure. The average potential is time-locked to the spike activity of the selected SMU. Our hypothesis stated that the resting SMU activity from the temporalis muscle contaminates the EEG records. Our study revealed the following original findings:

- Confirming our first hypothesis, some motor units in the temporalis are found to be spontaneously active and contaminate the EEG recordings.
- The contamination was location dependent and was most prominent on the side ipsilateral to the recorded SMU. However, interference was also notable in other electrodes that were further away from the SMU site and even in the contralateral side of the head.
- There was no time difference between the reflections of SMU on any of the EEG electrodes indicating cross-talk.
- Eyes open and eyes closed conditions did not significantly alter the interference of SMU activity on the EEG records.
- Jaw dropping activity stopped most of the spontaneously active units and hence may be used as a routine procedure in the future EEG studies.

### EMG activity at rest

In practice, a normal EEG recording condition refers to a state without any voluntary contraction of facial, neck, or masticatory muscles. Formerly, the EEG recorded at rest was assumed to contain a very low level of EMG (van de Velde et al., 1998) or be “EMG-free” (Akay and Daubenspeck, 1999; Fu et al., 2006). Goncharova et al. (2003) revealed the presence of considerable interference from the pericranial muscles to the EEG in the experimental rest condition. Similarly, recent paralysis studies confirmed the EMG contamination of EEG (Whitham et al., 2007; Pope et al., 2009; Fitzgibbon et al., 2013). Whitham et al. (2007) reported that the high frequency power which was quite evident around cranial and cervical muscles during resting un-paralyzed state declined significantly after paralysis in the frequencies above 20–30 Hz. Aligned with these previous findings, we demonstrated that some muscle activity continue around the head region under the “rest” conditions and contaminate the EEG recordings.

### SMU activity of temporalis

We recorded intramuscular EMG activity of temporalis instead of surface EMG which was the common technique in previous EEG studies (O'Donnell et al., 1974; Goncharova et al., 2003; Fu et al., 2006; Whitham et al., 2007; Yong et al., 2008; Fitzgibbon et al., 2013). According to the earlier work, the temporalis and frontalis muscles exhibited a low level of EMG activity even in the supine rest position (Jensen and Fuglsang-Frederiksen, 1994). Nonetheless, surface EMG is susceptible to the crosstalk from the neighboring muscles (Türker and Miles, 1990) and the limitation of it becomes more evident while measuring the resting activity of face musculature because of the anatomical proximity of facial and masticatory muscles. Consequently, we recorded intramuscular EMG activity from the anterior temporalis muscle (Figure 1A) under the physiological rest position of mandible which refers to a stable position of mandible relative to the maxilla (Nairn, 1976). Slight activity in the contractile elements of jaw elevators (masseter, temporalis and medial pterygoid muscles) are claimed to be responsible for the mandibular rest position (Møller, 1976; Rilo et al., 1976; Michelotti et al., 1997). Anatomically, the anterior part of the temporalis has the largest cross sectional area (Hannam and McMillan, 1994) and several intramuscular EMG studies revealed a specialized postural function for the anterior temporalis (Blanksma and van Eijden, 1990, 1995; McMillan, 1993; Blanksma et al., 1997). Furthermore, dense spindle population of temporalis (Kubota and Masegi, 1977) and predominance of type I fibers among type IIA, IIX, and hybrids (Korfage and van Eijden, 1999) indicate usage of anterior temporalis for tonic activities such as keeping the jaw position stable.

### Location dependence of the interference

In STA analysis, on average we have used 1000 triggers and hence cross-talk representations of the units are very reliable no matter where the recording electrodes were. Synchronous motor unit activity on the opposite side's temporalis muscle also cannot explain the contamination on the electrodes overlying the contralateral temporalis since synchronization is very weak even in the same jaw muscle (Nordstrom et al., 1990) and hence will be seen as noise in the averaged record.

The contamination (cross-talk) was location dependent and was most prominent on the site of the motor unit involved. Figure 2 presents the topography of Macro EMEGs in which frontal F7 and Fp1 electrodes expressed the highest contamination. Because of the spatial orientations of the fibers from which we recorded SMU, polarity and amplitude of the most prominent Macro-EMEGs differed. van Eijden et al. (1996) detailed the orientations of fiber bundles and the direction of pull of the anterior fibers. Around the intramuscular electrode, the pull angle of fibers inclined anteriorly and declined medially (van Eijden et al., 1996). Orientation of temporalis fibers evidences the clear phase reversal of the biphasic Macro-EMEGs when the average reference (AR) montage is preferred (Figures 2, 3).

**Figure 7:**
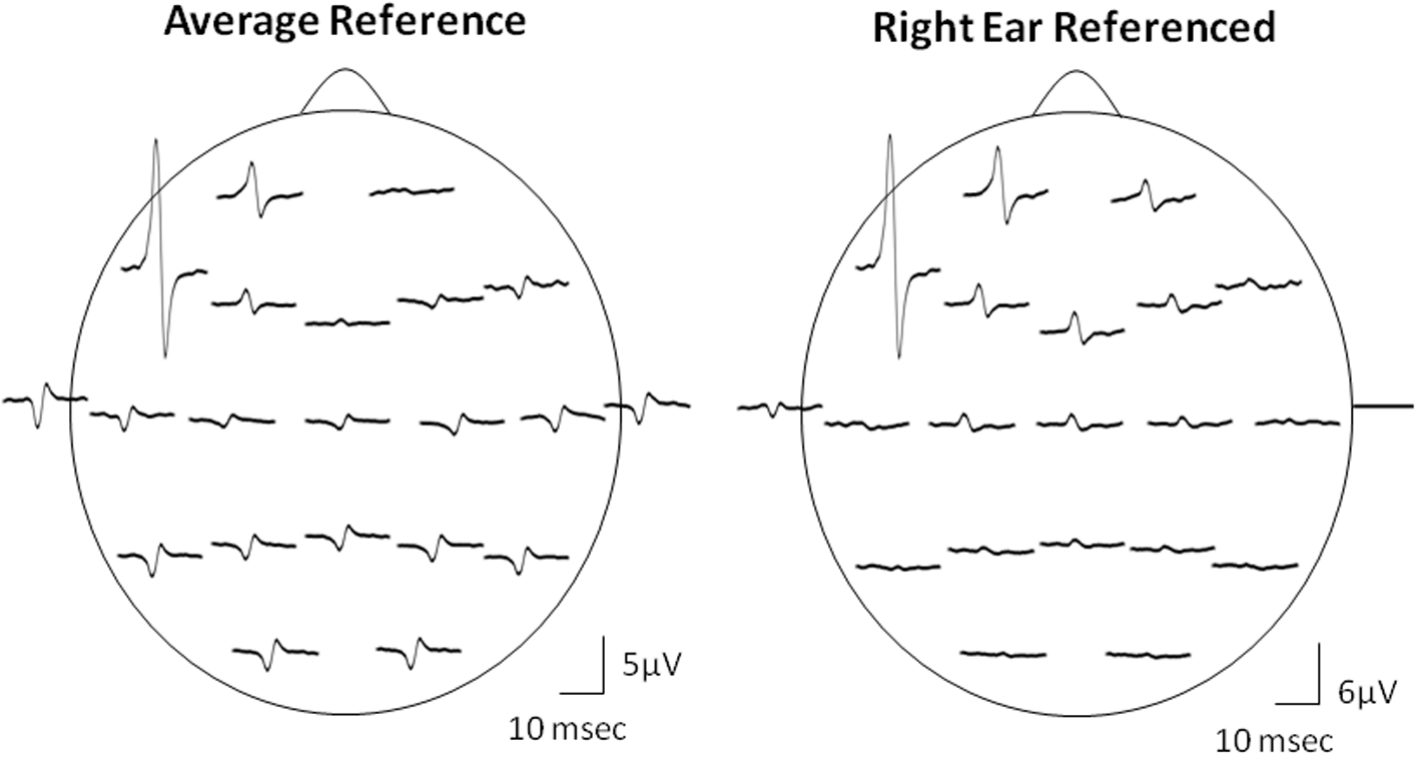
**Effect of referencing.** Comparison of AR and RE montages for the topography of Macro-EMEG representations from one SMU. Amplitude and polarity of potentials differ between the two references. The RE reference emphasized the anterior temporalis location more than the AR in terms of the amplitude of Macro-EMEGs. This comparison also indicates the fact that the phase-reversal and amplitude enhancements seen with AR at parietal and occipital sites were not genuine but due to distortion of the scalp topography of potentials by the referencing method.

Due to the “EEG reference problem” (Dien, 1998; Hagemann et al., 2001; Nunez and Srinivasan, 2006) different reference choices may result in different Macro-EMEG topographies. We preferred AR montage to demonstrate the interference of SMU on EEG. However, spatial distribution of the EEG electrodes is limited to the upper surface of the head and ideal situation for AR is when electrodes cover evenly the whole surface of the head (Nunez and Srinivasan, 2006). Figure 7 demonstrates the topographical difference between AR and right-ear (RE) reference. When RE reference was applied, the Macro-EMEG amplitudes were enhanced at frontal electrodes and they became smaller or disappeared completely at parietal and occipital electrodes. The observation that Macro-EMEG potentials were clearly pronounced on F7, F3 and Fp1 electrodes for both montages supports the hypothesis that interference from anterior temporalis is strongest around the recorded SMU independent to the reference used.

Global field power calculations resulted in no time delay; hence the reflections of SMU on EEG electrodes were synchronous, indicating cross-talk.

### Magnitude of the EMG interference

The magnitude of interference on each electrode location was compared by normalizing Macro-EMEG amplitudes to the percentage of the highest amplitude, either F7 or Fp1. Actual amplitude values of Macro-EMEG potentials varied slightly when measured in different episodes of the same SMU discharge. After percentage normalization, Macro-EMEGs showed similar percentage values for the same SMU. Figure 5 represents the magnitude of Macro-EMEG-potentials for different electrode locations. The SMU-trains belonging to same SMU had similar proportional distributions (Figure 5). The most dramatic difference was observed for the F7, Fp1, F3, and T3 due to the anatomical organization of temporalis fibers.

### Visible SMUs on EEG signal

During recording we observed visually distinct, irregular and regular rhythmical spikes in the EEG epochs which disappeared and appeared occasionally. Spike pattern was similar to the previously described “common ‘noise-like’ pattern” “railroad cross-tie pattern” and “beta rhythm-like pattern” (Goncharova et al., 2003; Ma et al., 2012). In Figure 4 SMU activity of temporalis appeared on F7 channel synchronously, however, activity on the T3 channel originated from a different fiber, asynchronous to the SMU followed. Not only the ipsilateral side but also the contralateral side showed visible SMU activity in some subjects.

Our observation of the visible SMU interference at rest emphasizes two important issues: comfort of the subject and the sampling regimen of the EEG signal. During recording, temporal, frontal, and occipital EEG channels reflected involuntary muscle activity, parallel to some observations reported (Goncharova et al., 2003; Ma et al., 2012). However, salient muscle activity was mostly diminished on the screen after intentional relaxation of subjects with a specific instruction. Consistent with our observation, Ma et al. (2012) suggested using stressful muscle regions as a biofeedback tool so that subjects can intentionally relax.

Moreover, alertness and stress caused by mental tasks given to subjects might cause a tension in facial muscles. When measured in the rest position, the EMG activity of frontalis, corrugators superciili (Cohen et al., 1992; Waterink and van Boxtel, 1994), and orbicularis oris inferior (Waterink and van Boxtel, 1994) increased with mental effort. The interference from head muscles to the EEG is a critical point for the studies measuring cognitive function involving different mental tasks. For example, the reliability of gamma band research is now under question in terms of the contribution of scalp muscle activity to EEG (Michel and Murray, 2009; Pope et al., 2009; Kovach et al., 2011; Nottage et al., 2013). In this study we showed that the term “rest” can be misleading because involuntary SMU activity from the temporalis continues at rest. A comprehensive investigation of SMU activity of masticatory, neck and facial muscles is an important need to evaluate muscle interference more reliably and to assess its possible effects on EEG more accurately.

EMG interference may not be considered as an issue in ERP (event-related potential) studies because this technique is based on “event-time-locked” averaging of a large number of EEG epochs. It should pose a serious problem, however, in ERO (event-related oscillation) research in which spectral components of “single” EEG sweeps or their average power spectra are evaluated but without any constraint of phase consistency.

Nine of 17 SMUs continued to fire after closing the eyes (Table 1). A comparison of the eyes open and closed conditions did not reveal a consistent change in Macro-EMEG amplitudes. However, eyes affected the relaxation of the subject and salient spikes on EEG channels were silenced remarkably after eye closing (Figure 6). We also observed that after subjects intentionally dropped their jaws, active SMUs stopped firing within 1–2 s. Therefore, similar instructions might be utilized in the EEG studies in an effort to minimize myogenic artifacts.

### Filtering and sampling of the EEG signal

The single motor unit action potential spikes contain high frequency components; therefore, the sampling rate for the SMU recordings should be high since inadequate sampling of the high frequency signals can distort their shape (Türker, 1993). We recorded SMU activity with a sampling rate of 4096 Hz. This rate was much higher than the rates used in EEG studies where EMG contamination was investigated (lower than 1000 Hz; van de Velde et al., 1998; Goncharova et al., 2003; Fu et al., 2006; Yong et al., 2008; Ma et al., 2012). With the high sampling rates we used, we succeeded to record clear SMU spikes from the temporalis muscles of six subjects.

In routine EEG studies, the band-pass filters are usually set to 0.1 Hz high-pass and 35 Hz or 70 Hz low-pass (Niedermeyer, 1999). Such low-pass filtering reduces the amplitude and the frequency content of the SMU spike activity dramatically, hence *making it appear like an EEG wave* (see Figure 1B). We therefore strongly recommend using high sampling rates and high levels of LP filtering (at least 1500 Hz) for recording and evaluation of the EEG signal. This will make sure that the SMU activity will be visible on the spike trigger averaged EEG records so that one can create special algorithms to handle these artifacts.

## Conclusion

Our study examined the interference of the resting temporalis activity on the EEG records. The contamination was dependent on the location of the EEG electrode relative to the recorded SMU and therefore was most prominent around the area of left anterior temporalis. Both sides of the head were contaminated, but the ipsilateral side received the strongest contaminations.

Even though we did not find a significant difference between the eyes open and closed states regarding the interference from the SMUs, it was evident that the some SMUs were silenced in the eyes closed condition. We recommend that the EEG should be recorded in eyes closed and jaw dropped conditions to decrease the amount of contamination from the resting activity of the jaw muscles and to generate a genuine relaxation of the subject. This measure is especially crucial in cases where “single” sweeps of EEG are analyzed and evaluated in time and frequency domains; e.g., in studies based on event related oscillations. This study may open a new path in evaluating the EMG contamination of the EEG and put further emphasis on the need for making the SMU spikes visible on the EEG so that efficient artifact removal techniques can be developed and used online to prevent such interferences.

### Conflict of interest statement

The authors declare that the research was conducted in the absence of any commercial or financial relationships that could be construed as a potential conflict of interest.
